# Tanshinone IIA inhibits metastasis after palliative resection of hepatocellular carcinoma and prolongs survival in part via vascular normalization

**DOI:** 10.1186/1756-8722-5-69

**Published:** 2012-11-08

**Authors:** Wen-Quan Wang, Liang Liu, Hui-Chuan Sun, Yan-Ling Fu, Hua-Xiang Xu, Zong-Tao Chai, Qiang-Bo Zhang, Ling-Qun Kong, Xiao-Dong Zhu, Lu Lu, Zheng-Gang Ren, Zhao-You Tang

**Affiliations:** 1 Liver Cancer Institute, Zhongshan Hospital, Fudan University; Key Laboratory for Carcinogenesis & Cancer Invasion (Fudan University), the Chinese Ministry of Education, 136 Yi Xue Yuan Road, Shanghai 200032, China; 2 Department of Pancreatic and Hepatobiliary Surgery, Fudan University Shanghai Cancer Center; Department of Oncology, Shanghai Medical College, Fudan University; Pancreatic Cancer Institute, Fudan University, 270 Dong An Road, Shanghai 200032, China; 3 Department of Neurology, Tongji Hospital, Tongji University, 390 Xin Cun Road, Shanghai 200032, China

**Keywords:** Tanshinone IIA, Vascular normalization, Palliative resection, Hepatocellular carcinoma, Metastasis

## Abstract

**Background:**

Promotion of endothelial normalization restores tumor oxygenation and obstructs tumor cells invasion, intravasation, and metastasis. We therefore investigated whether a vasoactive drug, tanshinone IIA, could inhibit metastasis by inducing vascular normalization after palliative resection (PR) of hepatocellular carcinoma (HCC).

**Methods:**

A liver orthotopic double-tumor xenograft model in nude mouse was established by implantation of HCCLM3 (high metastatic potential) and HepG2 tumor cells. After removal of one tumor by PR, the effects of tanshinone IIA administration on metastasis, tumor vascularization, and survival were evaluated. Tube formation was examined in mouse tumor-derived endothelial cells (TECs) treated with tanshinone IIA.

**Results:**

PR significantly accelerated residual hepatoma metastases. Tanshinone IIA did not inhibit growth of single-xenotransplanted tumors, but it did reduce the occurrence of metastases. Moreover, it inhibited PR-enhanced metastases and, more importantly, prolonged host survival. Tanshinone IIA alleviated residual tumor hypoxia and suppressed epithelial-mesenchymal transition (EMT) in vivo; however, it did not downregulate hypoxia-inducible factor 1α (HIF-1α) or reverse EMT of tumor cells under hypoxic conditions in vitro. Tanshinone IIA directly strengthened tube formation of TECs, associated with vascular endothelial cell growth factor receptor 1/platelet derived growth factor receptor (VEGFR1/PDGFR) upregulation. Although the microvessel density (MVD) of residual tumor tissue increased after PR, the microvessel integrity (MVI) was still low. While tanshinone IIA did not inhibit MVD, it did dramatically increase MVI, leading to vascular normalization.

**Conclusions:**

Our results demonstrate that tanshinone IIA can inhibit the enhanced HCC metastasis associated with PR. Inhibition results from promoting VEGFR1/PDGFR-related vascular normalization. This application demonstrates the potential clinical benefit of preventing postsurgical recurrence.

## Background

Surgical resection is the most promising strategy for early-stage hepatocellular carcinoma (HCC); however, the 5-year risk of recurrence is as high as 70% [1]. The surgery is actually palliative resection (PR), owing to the existence of satellites and microvascular invasion, and these residual tumor nests can actually be stimulated to grow by the PR [2,3]. Although several treatments, such as interferon-alpha and sorafenib, have been proposed to diminish relapse [4,5], prometastatic side effects of these options have also been observed [6,7].

Residual tumor cells may stimulate angiogenesis, which is needed for tumor growth [8-10]. However, the resulting neovessels may be disordered and inefficiently perfused, resulting in hypoxic conditions [10,11]. Both abnormal endothelium and pericytes integrated into the capillary wall, along with deficient coverage, could be responsible for the vascular architectural abnormalities [12]. The resulting hypoxia creates a hostile tumor milieu in which tumor cells may migrate via intra- or extravasation through a leaky vessel [9,13]. In effect, surgery-induced hypoxia unfavorably impacts the prognosis of cancer patients by inducing angiogenesis [14]. Therefore, restoring oxygen supply via vascular normalization may reduce metastasis, even tumor growth. Mazzone et al. [13] showed that downregulation of the oxygen sensing molecule PHD2 can restore tumor oxygenation and inhibit metastasis via endothelial normalization, where endothelial cells form a protective phalanx that blocks metastasis. Although several methods have been shown experimentally to promote vessel remodeling, only seldom has any of them found use in the clinic [9,10,15,16].

Tanshinone IIA (Tan IIA) is an herbal monomer with a clear chemical structure, isolated from *Salvia miltiorrhiza*. In Chinese traditional medicine, *S. miltiorrhiza* is considered to promote blood circulation for removing blood stasis and improve microcirculation. Some of these effects could include vessel normalization. We have reported that an herbal formula, Songyou Yin, can attenuate HCC metastases [17], and *S. miltiorrhiza* is one of the five constituents of the formula [18]. Tan IIA exhibits direct vasoactive [19,20] and certain antitumor properties [21]. It is possible that Tan IIA may indirectly decrease metastasis in HCC following PR by promoting blood vessel normalization; however, there is to date no evidence supporting this hypothesis.

We aimed to identify inhibitory effects of Tan IIA on HCC metastasis for delineating a possible mechanism of action of the compound, with a main focus on tumor vessel maturity as a potential marker for evaluating Tan IIA treatment responses.

## Results

### PR-induced residual tumor growth and metastasis

**Figure 1:**
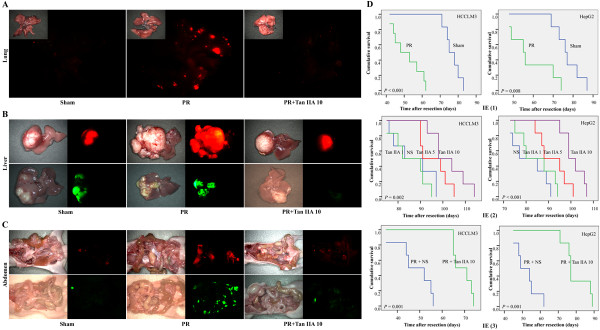
**Schematic diagram of metastases and cumulative survival of tumor**-**bearing nude mice in animal experiments. **Occurrence of lung (**A**), intrahepatic (**B**), and abdomen metastases (**C**) significantly increased after PR. Tan IIA decreased the numbers of various metastatic lesions. (**D**) Survival curves showed that the life-span of test mice was significantly shortened by PR and was markedly prolonged by Tan IIA. Red: HCCLM3. Green: HepG2.

As shown in the in vivo experiment 1 (IE 1) in Tables 1 and Additional file 1: Table S2, the tumor volume (TV) was greater in the PR than Sham groups (*p*<0.05, Additional file 1: Figure S1A). Compared with the Sham group, the lung metastasis (LM) (HCCLM3) of the PR group significantly increased (*p*<0.001, Figure 1A and S1B); both intrahepatic metastasis (IHM) and abdominal metastasis (AM) also increased (*p*<0.01 for IHM, in Figures 1B, Additional file 1: Figure S1C, and Additional file 1: Figure S2A; *p*<0.05 for AM, in Figures 1C, Additional file 1: Figure S1D, and Additional file 1: Figure S2B); and circulating tumor cells (CTCs) were elevated both at 2 and 35 d postresection (*p*<0.001, Additional file 1: Figure S1E).

**Table 1:** Summary of tumor growth, metastasis, and mice’s survival from three animal experiments of HCCLM3

**Items**	**IE (1)**			**IE (2)**					**IE (3)**		
	**Sham**	**PR**	***p***	**NS**	**Tan IIA 1**	**Tan IIA 5**	**Tan IIA 10**	***P***^**b**^	**PR+NS**	**PR+Tan IIA 10**	***p***
TV/cm^3^	2.712±1.262	5.990±3.113	.014	3.314±0.948	3.643±1.231	3.045±1.031	2.825±0.959	.268	4.736±1.660	3.339±0.924	.047
LM	7.22±4.71	110.11±28.01	.000^a^	7.42±3.61	6.25±2.77	4.83±3.01	2.25±2.86	.001	94.56±31.47	13.56±11.33	.000
CTCs	0.240±0.082	0.926±0.223	.000	ND	ND	ND	ND	–	0.966±0.276	0.283±0.093	.000^a^
35 d/%											
Survival/d	76.833±1.778	51.500±2.784	.000	87.000±3.804	85.500±3.128	96.000±2.658	102.667±3.201	.002	47.833±3.280	69.000±1.693	.001

^a^ Student’s *t*-test, equal variances assumed.

^b^ One-way analysis of variance.

Abbreviations: PR, palliative resection; NS, normal saline; CTCs, circulating tumor cells; IE, in vivo experiment; LM, lung metastasis; ND, not done; TV, tumor volume.

### Tan IIA does not directly inhibit tumor growth but reduces metastasis

Results of IE (2), shown in Tables 1 and Additional file 1: Table S2, indicate there were no differences in TV between the four groups observed (Additional file 1: Figure S1A). Compared with normal saline (NS), the LM of the Tan IIA-treated groups (5 or 10 mg/kg/d) decreased (*p*=0.046 and *p*<0.001, Additional file 1: Figure S1B). Compared with Tan IIA treatment of 1 or 5 mg/kg/d, the LM of the 10 mg/kg/d group also decreased (*p*=0.003 and *p*=0.046, Additional file 1: Figure S1B). Both the IHM and AM rates of the 5/10 mg Tan IIA/kg/d treatment groups were significantly reduced (*p*<0.05, Additional file 1: Figure S1C and D). No AM deriving from HepG2 cells was found. The greatest inhibitory effects were seen at a dosage of 10 mg/kg/d, which was chosen as the intervention dosage in IE (3).

### Tan IIA inhibits the PR-enhanced residual tumor metastasis

Results of IE (3), summarized in Tables 1 and Additional file 1: Table S2, show that administration of Tan IIA after PR resulted in decreased residual TV compared to NS (*p*<0.05, Additional file 1: Figure S1A). Compared with the PR + NS group, the LM (HCCLM3) of the Tan IIA treatment group was significantly decreased (*p*<0.001, Figures 1A and Additional file 1: Figure S1B); both IHM and AM also decreased (*p*<0.01 for IHM, Figures 1B, Additional file 1: Figure S1C, and S2A; *p*<0.01 for AM, Figures 1C, Additional file 1: Figure S1D, and S2B), and the CTCs were relatively decreased (*p*<0.001, Additional file 1: Figure S1E).

### Tan IIA prolongs host survival

Tan IIA treatment retarded the weight loss of mice after PR (Additional file 1: Figure S3). The estimated survival of PR mice was significantly shorter than of Sham mice in IE (1) (*p*<0.01, Tables 1 and Additional file 1: Table S2, Figure 1D). In IE (2), Tan IIA prolonged the mice’s survival up to 16 d for HCCLM3 (87.000 ± 3.804 vs. 102.667 ± 3.201, *p*=0.004) and 19 d for HepG2 (*p*<0.001, Tables 1 and Additional file 1: Table S2, Figure 1D), compared with NS. The same effect on prolongation of post-PR survival was seen in IE (3) (*p*=0.001 for both, Tables 1 and Additional file 1: Table S2, Figure 1D).

### Tan IIA does not inhibit proliferation but minimizes invasiveness of tumor cells

**Figure 2:**
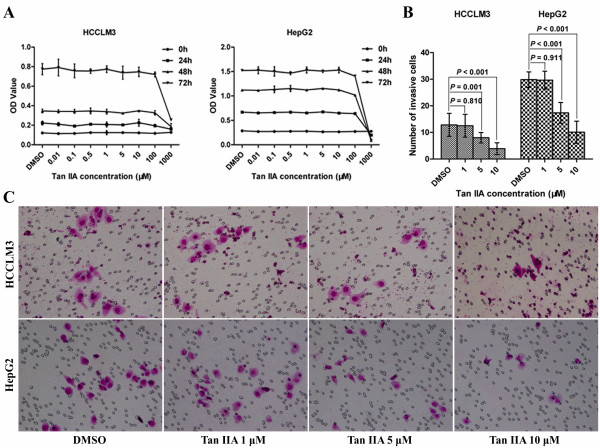
**Effects of Tan IIA on tumor cell proliferation and invasion. **(**A**) Tan IIA treatment at 0.01–100 μM did not inhibit HCCLM3 and HepG2 cell proliferation, except in the 1000-μM dosage group. (**B**), (**C**) Tan IIA treatment with 5 or 10 μM for 48 h inhibited tumor cell invasion (200×).

Compared with dimethylsulfoxide treatment control, the OD value of Tan IIA 0.01–100-μM treatment groups showed no change (Figure 2A). The number of invasive cells in the 5/10-μM Tan IIA groups was significantly reduced (*p*<0.01). No significant differences were seen for the 1-μM Tan IIA group (Figure 2B and C).

### Tan IIA alleviates residual tumor hypoxia in vivo but does not downregulate HIF-1 of tumor cells under hypoxic conditions in vitro

**Figure 3:**
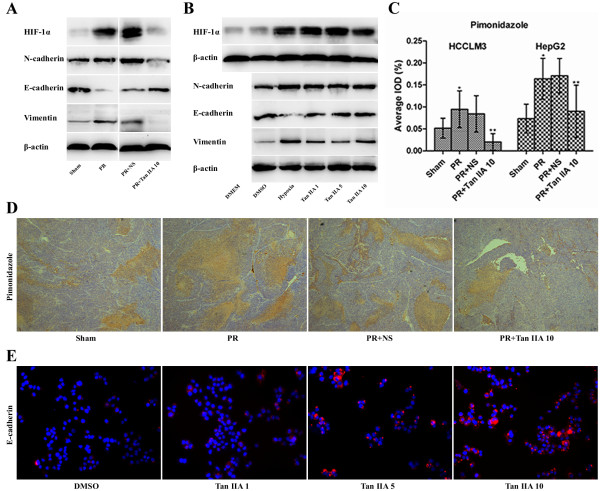
**Effects of Tan IIA on residual tumor, cell hypoxia and epithelial-mesenchymal transition. **(**A**) PR promoted HIF-1α expression and caused epithelial-mesenchymal transition (EMT; upregulated N-cadherin and vimentin, downregulated E-cadherin); Tan IIA reversed EMT in vivo. (**B**) Hypoxia-induced HIF-1α and promotion of EMT; Tan IIA had no effect on these parameters in vitro. (**C**), (**D**) Tan IIA diminished the enlarged Pimonidazole area of residual tumor after PR (50×). ^*^Compared with Sham group, ^**^compared with PR + NS group; *p*<0.001. (**E**) Tan IIA upregulated E-cadherin in tumor cells (200×).

The immunohistochemical marker for tissue hypoxia Pimonidazole and HIF-1α levels were significantly increased after PR, and they were reduced by Tan IIA. In addition, the residual tumor epithelial-mesenchymal transition (EMT) was enhanced (N-cadherin and Vimentin were both upregulated, but E-cadherin was downregulated), and this effect could be reversed by Tan IIA (Figures 3A,C, and D, and Additional file 1: Figure S4A). These results indicate that PR aggravated residual tumor hypoxia and promoted EMT, and Tan IIA treatment was able to alleviate hypoxia and inhibit EMT in vivo. Levels of HIF-1α, N-cadherin, and vimentin were upregulated in tumor cells, and E-cadherin was downregulated, under conditions of hypoxia. Other molecules were not downregulated as anticipated from results of Tan IIA experiments in vitro (Figures 3B and Additional file 1: Figure S4B). We did observe that E-cadherin expression could indeed be upregulated by Tan IIA, independent of the hypoxia effect (Figures 3E and Additional file 1: Figure S4C). Levels of proteins observed were consistent with their corresponding mRNA levels (Figures 3 and Additional file 1: Figure S4).

### Tan IIA does not affect microvessel density but promotes microvessel integrity

**Figure 4:**
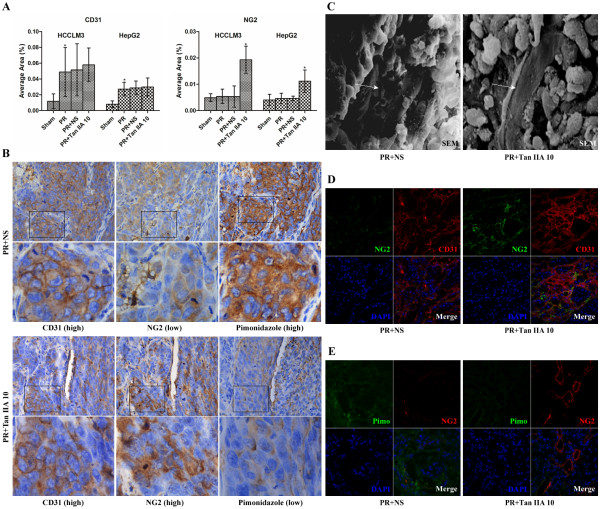
**Effects of Tan IIA on tumor microvessel density, microvessel integrity, and hypoxia. **(**A**) CD31 microvessel density (MVD) increased, and NG2 microvessel integrity (MVI) showed no change after PR. Tan IIA did not affect MVD, but it did elevate MVI. ^*^p<0.01. (**B**) PR + NS group: CD31 was high, NG2 was low, and Pimonidazole was high. PR + Tan IIA group: CD31 was high, NG2 was also high, and thus hypoxia was slight (400×). **(C) **The vascular wall of Tan IIA tumor tissue was more integrated than NS tissue (1200×). (**D**), (**E**) Tan IIA increased NG2 levels and reduced residual tumor hypoxia (200×).

Studies of mouse microvessel density (MVD) used the marker CD31. NG2 proteoglycan, the marker of vascular pericytes [12,15], was adopted to evaluate microvessel intensity (MVI). The CD31 levels of the PR group were higher than of the Sham (*p*<0.001), and there was no statistical significance between the PR + NS and Tan IIA groups (Figures 4A and Additional file 1: Figure S5A). Although the NG2 proteoglycan levels showed no change after PR, its level was significantly elevated in the PR + Tan IIA group (*p*<0.01, Figures 4A and Additional file 1: Figure S5B). Immunohistochemistry of CD31, NG2, and Pimonidazole in serial sections showed that in the PR + NS group, CD31 was high, NG2 was low, and the hypoxia levels (Pimonidazole) were seriously high. In the PR + Tan IIA group, CD31 was high, NG2 was also high, and hypoxia levels were slight (Figure 4B). These results indicate that the residual tumor MVD increased after PR, but the MVI was low. Tan IIA did not inhibit MVD but markedly improved MVI, promoting vessel integrity. Scanning electron microscopy (SEM) further revealed that the vascular wall of PR + Tan IIA tumors was more integrated than in the NS tumors (Figure 4C). Immunofluorescence (IF) results verified that Tan IIA did not affect CD31 levels, elevated NG2, and decreased hypoxia levels (Figure 4D and E).

### Tan IIA enhances tube formation and is associated with vascular endothelial cell growth factor receptor 1 (VEGFR1) and platelet derived growth factor receptor (PDGFR) upregulation

**Figure 5:**
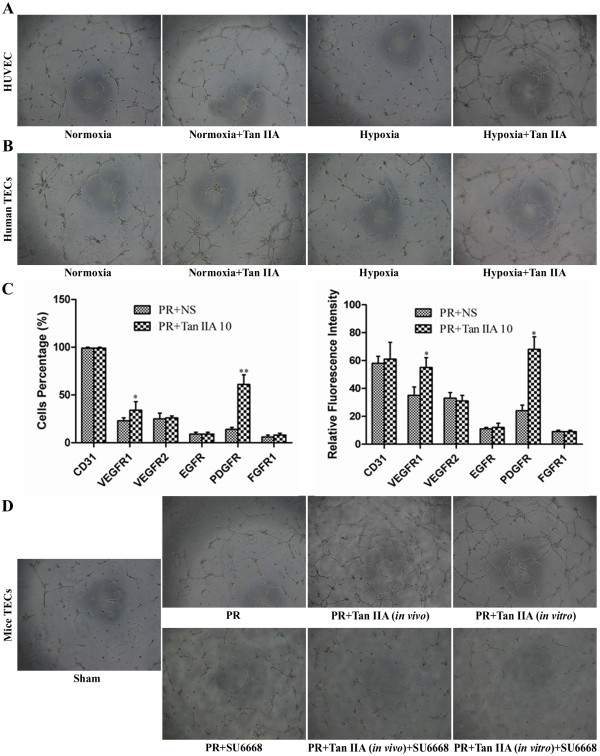
**Effects of Tan IIA on endothelial cells tube formation and TEC surface expression of growth factor receptors. **(**A**, **B**) Tan IIA enhanced tube formation of HUVECs and human TECs (50×). (**C**) The percentage of VEGFR1- and PDGFR-positive TECs and the relative fluorescence intensities. Values were higher in the PR + Tan IIA group than NS group. ^*^p<0.05, ^**^p<0.01. (**D**) Tube formation was enhanced by Tan IIA in vivo, similar to results seen with Tan IIA incubation in vitro; the addition of the VEGFR1/PDGFR inhibitor SU6668 weakened this enhancement effect.

Tube formation of human umbilical vein endothelial cells (HUVECs) and human tumor-derived endothelial cells (TECs) was enhanced by Tan IIA (Figure 5A and B). And tube formation of TECs from the PR + Tan IIA (in vivo) mouse group was strengthened relative to the PR group (Figure 5D). Using TECs from the PR group, we further found that tube formation was enhanced in vitro by Tan IIA, and it was roughly equivalent to the PR + Tan IIA (in vivo) group (Figure 5D). Subsequent flow cytometric analysis of VEGFR1 and PDGFR in TECs indicated that both the percentage of positive mice and relative cellular fluorescence intensities were significantly higher in the PR + Tan IIA group than the NS group (*p*<0.05), and no changes were seen in VEGFR2, EGFR, and FGFR1 levels (Figure 5C). Treatment of cells with the VEGFR1/PDGFR inhibitor SU6668 weakened the Tan IIA-dependent enhanced tube formation; whereas, low SU6668 concentration was not seen to inhibit tube formation without Tan IIA (Figure 5D).

## Discussion

In the present study of postsurgical residual tumors, we established a double-tumor xenograft HCC model and found that PR accelerated local aggressivity and distant metastasis. Administration of Tan IIA after PR significantly inhibited metastases and prolonged survival of nude mice bearing residual tumor tissue, and the effect was closely associated with VEGFR1/PDGFR-related vascular normalization.

Early in 1959, Fisher et al. [22] found that partial hepatectomy elicited hepatic metastases. Our results confirm the view that incomplete surgical resection of primary tumor may well induce the metastatic potential of residual tumor tissue. Currently, there is no evidence indicating that presurgical primary tumor could govern postsurgical residual tumor when growing in the same liver lobe. Interestingly, we found that residual tumor hypoxia was aggravated, HIF-1α was upregulated, and EMT was induced after surgical removal of “primary tumor.” Van der Bilt et al. [14] have reported that surgery-induced tumor hypoxia can stimulate abnormal angiogenesis. Our findings that neovascular abnormality is significantly augmented following PR are consistent with that report. Severe tumor hypoxia might have effects on abnormalities in the vasculature by promoting release of angiogenic cytokines [10], which would enhance metastasis.

Tumor vessel abnormalities could promote metastasis through mechanical penetration and hypoxia-induced EMT. We selected Tan IIA, known to possess potential vascular activity, to investigate inhibition of metastasis and the association with vascular normalization. We observed that Tan IIA could inhibit post-PR enhanced metastases and, more importantly, prolong survival. At a cellular level, Tan IIA showed no effect on tumor cell proliferation, but it minimized invasion. These effects were consistent with in vivo results showing that Tan IIA did not inhibit single-xenograft tumor growth but decreased metastases. A possible mechanism may be related by correlation with the observed E-cadherin upregulation. Furthermore, we observed that Tan IIA significantly alleviates residual tumor hypoxia and inhibits EMT in vivo. However, it did not downregulate HIF-1α or reverse EMT of tumor cells under hypoxic conditions in vitro. Therefore, we propose that the main inhibition of metastasis by Tan IIA is indirect.

We also observed that the tumor MVD increased after PR, but the MVI was low, suggesting that the surgery-induced angiogenesis was related to structural abnormalities [14]. Tumor cells would metastasize more easily through such a leaky vascular wall, causing an increase of CTCs. Tan IIA did not inhibit MVD but dramatically improved MVI, which is probably related to its underlying vasoactivity. Tan IIA also enhanced tube formation of endothelial cells under hypoxic conditions in vitro. Additionally, we found that tube formation of mouse TECs which processed with Tan IIA no matter in vivo or in vitro, was similar to each other, further indicating a direct effect of Tan IIA on endothelium. How Tan IIA may promote vascular normalization is not entirely clear because its receptor on endothelial cells is unknown. Our results have shown a possible correlation with VEGFR1 and PDGFR upregulation. Recently, it has been reported that inhibition of VEGFR1 and PDGFR signaling in several tumors causes pericyte detachment and vessel regression, leading to vascular abnormalities [23,24]. This implies that upregulation of this signaling might produce a beneficial effect. Of relevance is the observation that a compound AZD2171 inhibits VEGFR while promoting vessel normalization [16]. The mechanism of Tan IIA action requires further investigation.

Vascular normalization has effects on two major processes: (i) mechanical prevention of tumor cell migration via intra/extravasation; and (ii) restoration of oxygen and nutritional supply. However, recovery of tumor blood supply may have mixed effects by contributing to progression while also suppressing tumor growth [9]. The latter effect is likely to depend on a combination of factors. The normalization of vessels from both direct and indirect Tan IIA effects appears to be involved with inhibition of residual tumor growth, invasion, and metastasis.

## Conclusions

Our findings demonstrate that Tan IIA can inhibit the enhanced metastasis induced by PR and may do so in part via VEGFR1/PDGFR-related vascular normalization. This work has an important implication: that the malignant phenotype of a tumor may be manipulated through vascular pathways, which could be an alternative to simple eradication. Our results highlight the potential of proangiogenic “vessel normalizing” treatment strategies to silence metastasis and prolong patient survival.

## Methods

### Cell lines, animal model, and drug

The human HCC cell lines HCCLM3-RFP, which has high metastatic potential [25], and HepG2-GFP [26], HUVECs, and TECs [27] were used in the studies. Male, athymic BALB/c *nu/nu* mice of 4–6 weeks of age and weighing approximately 20 g were used as host animals.

A metastatic human HCC animal model was established by orthotopic implantation of histologically intact tumor tissue into the nude mouse liver [28]. To explore the protumoral effects of PR, we constructed an orthotopic double-tumor xenograft model, in which two tumor pieces were simultaneously inoculated into the left liver lobe; the inoculation method was as described [28]. After 2 weeks, partial hepatectomy [29] was performed to excise one tumor. The Sham hepatectomy cohort was handled like the PR cohort, but without tumor resection.

Tan IIA (sulfotanshinone sodium injection, 5 mg/ml), commercially available from the first Biochemical Pharmaceutical Co. Ltd., Shanghai, China, was used in *in vivo* experiment. Tan IIA monomer (Sigma, St. Louis, MO), a reddish lyophilized powder with the purity 99.99%, firstly dissolved in dimethyl sulfoxide and then diluted with NS to the required concentration, was used in *in vitro* study.

### Experimental groups and assessment parameters

For IE (1), 30 double-tumor-bearing mice were randomly divided into Sham and PR groups (each of *n*=15) and scheduled to be observed after 35 d. In IE (2), the single-tumor xenograft model was used. Mice were divided into four groups (each *n*=18) and received daily injections of NS or Tan IIA (1, 5, or 10 mg/kg/d). Tan IIA was diluted with NS. We took 20 g as the average mouse weight (25 g after 21 d), and each mouse received 0.2 mL solution intraperitoneally for 35 d. In IE (3), the double-tumor xenograft plus PR model was used to examine effects of Tan IIA on residual tumor. Mice were divided into two groups (each *n*=15) after PR and received daily injections of NS or 10 mg Tan IIA/kg/d for 35 d.

The mouse weight was measured once every 7 d. After 35 d, six mice from each group were retained to observe survival, and the remaining were sacrificed to measure TV [30], LM, IHM, AM [26], CTCs, and to perform SEM of tumor vessels. CTCs were enumerated by flow cytometry and expressed as percent CTCs/TV (%) [6]. Twelve mice (IE 1, *n* = 6) were sacrificed 2 d after resection to examine CTCs shortly after PR.

### Cell proliferation and invasion

A Cell Counting Kit-8 (Dojindo, Kumamoto, Japan) was used to assay cell proliferation. The final concentration of Tan IIA was 0.01–1000 μM. Results were expressed as OD at 490 nm. Cell invasiveness was assayed in Matrigel-coated Transwell Invasion Chambers (Corning, Cambridge, MA). Tan IIA was added to cells at final concentrations of 1, 5, or 10 μM, and these cultures were incubated for 48 h. Cells that passed through the chamber membranes were counted.

### Hypoxia evaluation

Cells were cultured in a Bugbox Hypoxic Workstation (Ruskinn, Mid Glamorgan, UK; 1% O_2_, 5% CO_2_, and 94% N_2_ atmosphere) and incubated with Tan IIA at 1, 5, or 10 μM for 48 h. Normoxic conditions (20% O_2_, 5% CO_2_, and 75% N_2_) were set as control. Pimonidazole immunostaining and HIF-1α expression were defined as hypoxia biomarkers. A Hypoxyprobe™-1 Kit (Hypoxyprobe Inc., Burlington, MA) was used [6].

### Isolation of TECs, flow cytometry, and tube formation

Eight tumors from Sham, PR, or PR + Tan IIA groups were collected. The TECs were isolated by use of anti-CD31 antibody (AB)-coupled magnetic beads (Miltenyi Biotec, Cologne, Germany) and magnetic cell-sorting system [27], and they were divided into Sham, PR, PR + Tan IIA (in vivo), and PR + Tan IIA (in vitro) groups; TECs isolated from the PR group were incubated with Tan IIA for 48 h. TEC surface expression of VEGFR1, VEGFR2, EGFR, PDGFR, FGFR1, and CD31 was determined by flow cytometric analysis (R&D, Minneapolis, MN). Receptor density was calculated as the relative fluorescence intensity. In another set of experiments, TECs from the PR + Tan IIA (in vivo) cohort were divided into control and SU6668 (Sigma) (VEGFR1/PDGFR selective receptor inhibitor) treatment groups. TECs from the PR cohort were also divided into PR, PR + SU6668, PR + Tan IIA (in vitro), and PR + SU6668 + Tan IIA groups. HUVECs and human TECs were separated into control, normoxia + Tan IIA, and hypoxia + Tan IIA groups. Formation of capillary-like structures was observed as described [27].

### Immunohistochemistry, immunofluorescence, western blot, and quantitative real-time polymerase chain reaction

Immunohistochemistry [31] of Pimonidazole, CD31, and NG2 [15] was performed in paraffin sections on slides. The primary antibodies to Pimonidazole (1:100), CD31 (1:100; Abcam, Cambridge, MA), and NG2 (1:200; Millipore, Billerica, MA) were selected. The integrated optical density (IOD; for Pimonidazole) or area (for CD31 and NG2) of positive staining/total area was quantified by Image-Pro Plus software [31]. IF double-staining [31] of CD31 (1:50) and NG2 (1:50), and NG2 and Pimonidazole (1:80) was done in frozen sections and observed under laser confocal microscope. IF of E-cadherin in cells was also determined.

Protein levels of HIF-1α, N-cadherin, E-cadherin, and vimentin were determined by immunoblot analysis. Primary antibodies against HIF-1α (1:1000; Sigma), β-actin, N-cadherin (1:1000; Abcam), E-cadherin (1:400; Santa Cruz Biotechnology, Santa Cruz, CA), and vimentin (1:800; Cell Signaling Technology, Beverly, MA) were used. Levels of mRNA were assessed by polymerase chain reaction (Additional file 1: Table S1) and normalized to the corresponding internal β-actin signal (ΔCt). Relative gene expression values were expressed as 2^−ΔΔCt^[30].

### Statistical analysis

All statistical analyses were performed with the SPSS 16.0 software. The Pearson chi-square test was applied to compare qualitative variables. Quantitative variables were expressed as mean ± standard deviations and analyzed by *t*-test or one-way analysis of variance followed by least significant difference test. The Kaplan–Meier method with log-rank test was used for survival analysis. A *p* value of <0.05 was considered to be statistically significant.

### Ethics approval

Animal care and experimental protocols were approved by the Shanghai Medical Experimental Animal Care Commission.

## Abbreviations

AM: Abdominal metastasis; CTCs: Circulating tumor cells; EMT: Epithelial-mesenchymal transition; HCC: Hepatocellular carcinoma; HUVEC: Human umbilical vein endothelial cells; IE: In vivo experiment; IF: Immunofluorescence; IHM: Intrahepatic metastasis; IOD: Integrated optical density; LM: Lung metastasis; MVD: Microvessel density; MVI: Microvessel integrity; NS: Normal saline; PDGFR: Platelet derived growth factor receptor; PR: Palliative resection; SEM: Scanning electron microscopy; Tan IIA: Tanshinone IIA; TEC: Tumor-derived endothelial cells; TV: Tumor volume; VEGFR1: Vascular endothelial cell growth factor receptor 1.

## Competing interests

The authors declare there are no competing interests.

## Authors’ contributions

WWQ designed the study, established the animal model, carried out the immunoassays, performed the statistical analysis, and drafted the manuscript. LL and SHC participated in the design of the study, data analysis, and drafting the manuscript. FYL, XHX, CZT, ZQB, KLQ, ZXD, and LL helped to acquire experimental data. RZG reviewed the manuscript. TZY conceived the study, participated in its design and coordination, and helped to draft the manuscript. All authors read and approved the final manuscript.

## Authors’ information

WWQ, M.D., Ph.D., in Cancer Surgery. Graduated from Liver Cancer Institute, Zhongshan Hospital, Fudan University; Key Laboratory for Carcinogenesis & Cancer Invasion (Fudan University), the Chinese Ministry of Education. WWQ is now working in the Department of Pancreatic and Hepatobiliary Surgery, Fudan University, Shanghai Cancer Center; the Department of Oncology, Shanghai Medical College, Fudan University; and the Pancreatic Cancer Institute, Fudan University.

## Supplementary Material

Additional file 1 **Table S1. **The primer sequences for amplification of human HIF-1α, N-cadherin, E-cadherin, Vimentin, and β-actin. **Table S2. **Summary of tumor growth, metastasis, and survival of host mice in three animal experiments. **Figure S1. **Statistical chart of tumor volume **(A)**, metastases to lung **(B)**, intrahepatic **(C)**, and to abdomen **(D)**, and circulating tumor cells **(E) **in three animal experiments. ^*^For Student’s *t*-test, equal variances were assumed. Abbreviations: CTCs, circulating tumor cells; IHM, intrahepatic metastasis. **Figure S2. **Schematic diagram of intrahepatic and abdomen metastases. **(A)** Intrahepatic metastatic lesions were shown by HE staining (50×). **(B) **Typical abdomen metastasis of residual HCCLM3 tumor 35 d after palliative resection. Panels a and b show intrahepatic, peritoneal, and diaphragmatic metastatic lesions. c and d show intrahepatic and diaphragmatic metastatic lesions; d shows the area where the tumor in c was removed. **Figure S3.** Monitoring body weight of experimental mice in three in vivo experiments. **(A) **In vitro experiment 1 (IE 1). **(B) **IE (2). **(C) **IE (3). **Figure S4. **Intratumoral and intracellular mRNA levels of HIF-1α, N-cadherin, E-cadherin, and Vimentin. (A)^*^Compared with Sham group, ^**^compared with PR + NS group; *p*<0.05. (B)^*^Compared with dimethylsulfoxide (DMSO) group, ^**^compared with hypoxia group; *p*<0.05. (C)^*^Compared with DMSO group; *p*<0.05. **Figure S5. **Immunohistochemical staining of CD31 **(A) **and NG2 **(B) **in tumor samples. Click here for file

## References

[B1] El-Serag HB Hepatocellular carcinoma N Engl J Med 2011 365 1118 1127 10.1056/NEJMra1001683 21992124

[B2] Meredith K Haemmerich D Qi C Mahvi D Hepatic resection but not radiofrequency ablation results in tumor growth and increased growth factor expression Ann Surg 2007 245 771 776 10.1097/01.sla.0000261319.51744.59 17457170 PMC1877067

[B3] Takemoto Y Li TS Kubo M Ohshima M Ueda K Harada E Enoki T Okamoto M Mizukami Y Murata T Hamano K Operative injury accelerates tumor growth by inducing mobilization and recruitment of bone marrow-derived stem cells Surgery 2011 149 792 800 10.1016/j.surg.2011.02.005 21507448

[B4] Wang L Tang ZY Qin LX Wu XF Sun HC Xue Q Ye SL High-dose and long-term therapy with interferon-alfa inhibits tumor growth and recurrence in nude mice bearing human hepatocellular carcinoma xenografts with high metastatic potential Hepatology 2000 32 43 48 10869287 10.1053/jhep.2000.8525

[B5] Feng YX Wang T Deng YZ Yang P Li JJ Guan DX Yao F Zhu YQ Qin Y Wang H Sorafenib suppresses postsurgical recurrence and metastasis of hepatocellular carcinoma in an orthotopic mouse model Hepatology 2011 53 483 492 10.1002/hep.24075 21274870

[B6] Zhuang PY Zhang JB Zhang W Zhu XD Liang Y Xu HX Xiong YQ Kong LQ Wang L Wu WZ Long-term interferon-alpha treatment suppresses tumor growth but promotes metastasis capacity in hepatocellular carcinoma J Cancer Res Clin Oncol 2010 136 1891 1900 10.1007/s00432-010-0848-1 20213095 PMC11828181

[B7] Ebos JM Lee CR Cruz-Munoz W Bjarnason GA Christensen JG Kerbel RS Accelerated metastasis after short-term treatment with a potent inhibitor of tumor angiogenesis Cancer Cell 2009 15 232 239 10.1016/j.ccr.2009.01.021 19249681 PMC4540346

[B8] Al-Sahaf O Wang JH Browne TJ Cotter TG Redmond HP Surgical injury enhances the expression of genes that mediate breast cancer metastasis to the lung Ann Surg 2010 252 1037 1043 10.1097/SLA.0b013e3181efc635 21107114

[B9] Rolny C Mazzone M Tugues S Laoui D Johansson I Coulon C Squadrito ML Segura I Li X Knevels E HRG inhibits tumor growth and metastasis by inducing macrophage polarization and vessel normalization through downregulation of PlGF Cancer Cell 2011 19 31 44 10.1016/j.ccr.2010.11.009 21215706

[B10] Jain RK Normalization of tumor vasculature: an emerging concept in antiangiogenic therapy Science 2005 307 58 62 10.1126/science.1104819 15637262

[B11] Goel S Duda DG Xu L Munn LL Boucher Y Fukumura D Jain RK Normalization of the vasculature for treatment of cancer and other diseases Physiol Rev 2011 91 1071 1121 10.1152/physrev.00038.2010 21742796 PMC3258432

[B12] Raza A Franklin MJ Dudek AZ Pericytes and vessel maturation during tumor angiogenesis and metastasis Am J Hematol 2010 85 593 598 10.1002/ajh.21745 20540157

[B13] Mazzone M Dettori D Leite De Oliveira R Loges S Schmidt T Jonckx B Tian YM Lanahan AA Pollard P Ruiz De Almodovar C Heterozygous deficiency of PHD2 restores tumor oxygenation and inhibits metastasis via endothelial normalization Cell 2009 136 839 851 10.1016/j.cell.2009.01.020 19217150 PMC4037868

[B14] van der Bilt JD Borel Rinkes IH Surgery and angiogenesis Biochim Biophys Acta 2004 1654 95 104 14984770 10.1016/j.bbcan.2004.01.003

[B15] Sasajima J Mizukami Y Sugiyama Y Nakamura K Kawamoto T Koizumi K Fujii R Motomura W Sato K Suzuki Y Transplanting normal vascular proangiogenic cells to tumor-bearing mice triggers vascular remodeling and reduces hypoxia in tumors Cancer Res 2010 70 6283 6292 10.1158/0008-5472.CAN-10-0412 20631070 PMC3063898

[B16] Batchelor TT Sorensen AG di Tomaso E Zhang WT Duda DG Cohen KS Kozak KR Cahill DP Chen PJ Zhu M AZD2171, a pan-VEGF receptor tyrosine kinase inhibitor, normalizes tumor vasculature and alleviates edema in glioblastoma patients Cancer Cell 2007 11 83 95 10.1016/j.ccr.2006.11.021 17222792 PMC2748664

[B17] Huang XY Huang ZL Wang L Xu YH Ai KX Zheng Q Tang ZY Herbal compound "Songyou Yin" reinforced the ability of interferon-alfa to inhibit the enhanced metastatic potential induced by palliative resection of hepatocellular carcinoma in nude mice BMC Cancer 2010 10 580 10.1186/1471-2407-10-580 20969807 PMC2976755

[B18] Huang XY Wang L Huang ZL Zheng Q Li QS Tang ZY Herbal extract "Songyou Yin" inhibits tumor growth and prolongs survival in nude mice bearing human hepatocellular carcinoma xenograft with high metastatic potential J Cancer Res Clin Oncol 2009 135 1245 1255 10.1007/s00432-009-0566-8 19277711 PMC12160221

[B19] Wang J Dong MQ Liu ML Xu DQ Luo Y Zhang B Liu LL Xu M Zhao PT Gao YQ Li ZC Tanshinone IIA modulates pulmonary vascular response to agonist and hypoxia primarily via inhibiting Ca2+ influx and release in normal and hypoxic pulmonary hypertension rats Eur J Pharmacol 2010 640 129 138 10.1016/j.ejphar.2010.04.047 20460121

[B20] Fan G Zhu Y Guo H Wang X Wang H Gao X Direct vasorelaxation by a novel phytoestrogen tanshinone IIA is mediated by nongenomic action of estrogen receptor through endothelial nitric oxide synthase activation and calcium mobilization J Cardiovasc Pharmacol 2011 57 340 347 10.1097/FJC.0b013e31820a0da1 21383591

[B21] Wang L Zhou GB Liu P Song JH Liang Y Yan XJ Xu F Wang BS Mao JH Shen ZX Dissection of mechanisms of Chinese medicinal formula Realgar-Indigo naturalis as an effective treatment for promyelocytic leukemia Proc Natl Acad Sci U S A 2008 105 4826 4831 10.1073/pnas.0712365105 18344322 PMC2290784

[B22] Fisher B Fisher ER Experimental studies of factors influencing hepatic metastases, III. Effect of surgical trauma with special reference to liver injury Ann Surg 1959 150 731 744 10.1097/00000658-195910000-00015 13823186 PMC1613460

[B23] Bergers G Song S Meyer-Morse N Bergsland E Hanahan D Benefits of targeting both pericytes and endothelial cells in the tumor vasculature with kinase inhibitors J Clin Invest 2003 111 1287 1295 12727920 10.1172/JCI17929 PMC154450

[B24] Erber R Thurnher A Katsen AD Groth G Kerger H Hammes HP Menger MD Ullrich A Vajkoczy P Combined inhibition of VEGF and PDGF signaling enforces tumor vessel regression by interfering with pericyte-mediated endothelial cell survival mechanisms FASEB J 2004 18 338 340 14657001 10.1096/fj.03-0271fje

[B25] Li Y Tang ZY Hou JX Hepatocellular carcinoma: insight from animal models Nat Rev Gastroenterol Hepatol 2011 9 32 43 10.1038/nrgastro.2011.196 22025031

[B26] Yang BW Liang Y Xia JL Sun HC Wang L Zhang JB Tang ZY Liu KD Chen J Xue Q Biological characteristics of fluorescent protein-expressing human hepatocellular carcinoma xenograft model in nude mice Eur J Gastroenterol Hepatol 2008 20 1077 1084 10.1097/MEG.0b013e3283050a67 19047839

[B27] Xiong YQ Sun HC Zhang W Zhu XD Zhuang PY Zhang JB Wang L Wu WZ Qin LX Tang ZY Human hepatocellular carcinoma tumor-derived endothelial cells manifest increased angiogenesis capability and drug resistance compared with normal endothelial cells Clin Cancer Res 2009 15 4838 4846 10.1158/1078-0432.CCR-08-2780 19638466

[B28] Sun FX Tang ZY Lui KD Ye SL Xue Q Gao DM Ma ZC Establishment of a metastatic model of human hepatocellular carcinoma in nude mice via orthotopic implantation of histologically intact tissues Int J Cancer 1996 66 239 243 10.1002/(SICI)1097-0215(19960410)66:2<239::AID-IJC17>3.0.CO;2-7 8603818

[B29] Rashidi B An Z Sun FX Li X Tang ZY Moossa AR Hoffman RM Efficacy of intra-hepatectomy 5-FU on recurrence and metastasis of human hepatocellular carcinoma in nude mice Int J Cancer 2001 91 231 235 10.1002/1097-0215(200002)9999:9999<::AID-IJC1042>3.3.CO;2-O 11146450

[B30] Jia JB Wang WQ Sun HC Liu L Zhu XD Kong LQ Chai ZT Zhang W Zhang JB Xu HX A novel tripeptide, tyroserleutide, inhibits irradiation-induced invasiveness and metastasis of hepatocellular carcinoma in nude mice Invest New Drugs 2011 29 861 872 10.1007/s10637-010-9435-1 20414698

[B31] Jia JB Wang WQ Sun HC Zhu XD Liu L Zhuang PY Zhang JB Zhang W Xu HX Kong LQ High expression of macrophage colony-stimulating factor-1 receptor in peritumoral liver tissue is associated with poor outcome in hepatocellular carcinoma after curative resection Oncologist 2010 15 732 743 10.1634/theoncologist.2009-0170 20551429 PMC3228006

