# Supplementary material for: Tanshinone IIA inhibits metastasis after palliative resection of hepatocellular carcinoma and prolongs survival in part via vascular normalization
Source: J Hematol Oncol. 2012 Nov 8;5:69. doi: 10.1186/1756-8722-5-69 (PMC3506473; doi:10.1186/1756-8722-5-69)
Supplement: Additional file 1 — Table S1. The primer sequences for amplification of human HIF-1α, N-cadherin, E-cadherin, Vimentin, and β-actin. Table S2. Summary of tumor growth, metastasis, and survival of host mice in three animal experiments. Figure S1. Statistical chart of tumor volume (A), metastases to lung (B), intrahepatic (C), and to abdomen (D), and circulating tumor cells (E) in three animal experiments. *For Student’s t-test, equal variances were assumed. Abbreviations: CTCs, circulating tumor cells; IHM, intrahepatic metastasis. Figure S2. Schematic diagram of intrahepatic and abdomen metastases. (A) Intrahepatic metastatic lesions were shown by HE staining (50×). (B) Typical abdomen metastasis of residual HCCLM3 tumor 35 d after palliative resection. Panels a and b show intrahepatic, peritoneal, and diaphragmatic metastatic lesions. c and d show intrahepatic and diaphragmatic metastatic lesions; d shows the area where the tumor in c was removed. Figure S3. Monitoring body weight of experimental mice in three in vivo experiments. (A) In vitro experiment 1 (IE 1). (B) IE (2). (C) IE (3). Figure S4. Intratumoral and intracellular mRNA levels of HIF-1α, N-cadherin, E-cadherin, and Vimentin. (A)*Compared with Sham group, **compared with PR + NS group; p<0.05. (B)*Compared with dimethylsulfoxide (DMSO) group, **compared with hypoxia group; p<0.05. (C)*Compared with DMSO group; p<0.05. Figure S5. Immunohistochemical staining of CD31 (A) and NG2 (B) in tumor samples. [file 1756-8722-5-69-S1.doc]

**Supplementary data:**

**Table S1. The primer sequences for amplification of human HIF-1α, N-cadherin, E-cadherin, Vimentin, and β-actin**

| HIF-1α | forward | 5′-GCTGACCCTGCACTCAAT-3′ |
| --- | --- | --- |
|  | reverse | 5′-GATCGAAGGAAGGTAACTGG-3′ |
| N-cadherin | forward | 5′-CCGGAGAACAGTCTCCAACTC-3′ |
|  | reverse | 5′-CCCACAAAGAGCAGCAGTC-3′ |
| E-cadherin | forward | 5′-TGCCCAGAAAATGAAAAAGG-3′ |
|  | reverse | 5′-GTGTATGTGGCAATGCGTTC-3′ |
| Vimentin | forward | 5′-CGACAAGGTGCGCTTCCT-3′ |
|  | reverse | 5′-CCTGGCCCTTGAGCTGC-3′ |
| β-actin | forward | 5′-CATCTCTTGCTCGAAGTCCA-3′ |
|  | reverse | 5′-ATCATGTTTGAGACCTTCAACA-3′ |

**Table S2. Summary of tumor growth, metastasis, and survival of host mice in three animal experiments**

| HCCLM3 | Items | IE (1) | | | IE (2) | | | | | IE (3) | | |
| --- | --- | --- | --- | --- | --- | --- | --- | --- | --- | --- | --- | --- |
|  |  | Sham | PR | *p* | NS | Tan IIA 1 | Tan IIA 5 | Tan IIA 10 | *P*^b^ | PR+NS | PR+Tan IIA 10 | *p* |
|  | IHM | 1.56± 1.13 | 9.44± 5.77 | .001^a^ | 8/12 | 7/12 | 4/12 | 2/12 | .053 | 9.00±  3.87 | 2.22±  1.72 | .000^a^ |
|  | AM | 23.00±15.02 | 93.11±41.14 | .000^a^ | 12.17±8.29 | 12.92±  7.20 | 6.33±  5.74 | 2.25±  3.11 | .000 | 99.67±  29.75 | 12.22±  16.12 | .000 |
|  | CTCs  2 d/% | 0.810±0.155 | 3.786± 0.865 | .000^a^ | ND | ND | ND | ND | – | ND | ND | – |
| HepG2 | TV/cm^3^ | 2.094±0.775 | 5.041±3.368 | .031 | 2.942±0.542^c^ | 2.469±  0.585 | 2.633±  1.016^c^ | 2.311±  0.611 | .319 | 3.978±  0.716 | 2.963±  1.070 | .033 |
|  | LM | NF | NF | – | NF | NF | NF | NF | – | NF | NF | – |
|  | IHM | 1.78± 0.97 | 5.56± 1.59 | .000 | 7/8 | 7/9 | 3/8 | 2/9 | .017 | 6.00±  2.50 | 1.67±  1.23 | .001 |
|  | AM | 0.11± 0.33 | 20.56±22.88 | .016^a^ | NF | NF | NF | NF | – | 21.67±  18.64 | 2.56±  3.91 | .008^a^ |
|  | CTCs  2 d/% | 0.912± 0.168 | 4.134± 0.917 | .000^a^ | ND | ND | ND | ND | – | ND | ND | – |
|  | CTCs  35 d/% | 0.286± 0.086 | 1.017± 0.300 | .000^a^ | ND | ND | ND | ND | – | 0.998±  0.234 | 0.332±  0.095 | .000^a^ |
|  | Survival/d | 77.333±2.616 | 58.667±4.440 | .008 | 81.500±3.202 | 84.833  ±3.240 | 92.000  ±2.708 | 101.333  ±1.874 | .000 | 52.167  ±2.496 | 79.667  ±2.940 | .001 |

^a^Student’s *t*-test, equal variances assumed.

^b^One-way analysis of variance.

^c^A mouse was found dead unexpectedly during administration.

Abbreviations: AM, abdomen metastasis; CTCs, circulating tumor cells; IE, *in vivo* experiment; IHM, intrahepatic metastasis; LM, lung metastasis; ND, not done; NF, not found; PR, palliative resection; TV, tumor volume.

**Supplemental figure legends:**

**Figure S1. Statistical chart of tumor volume (A), metastases to lung (B), intrahepatic (C), and to abdomen (D), and circulating tumor cells (E) in three animal experiments.** ^*^For Student’s *t*-test, equal variances were assumed. Abbreviations: CTCs, circulating tumor cells; IHM, intrahepatic metastasis.

**
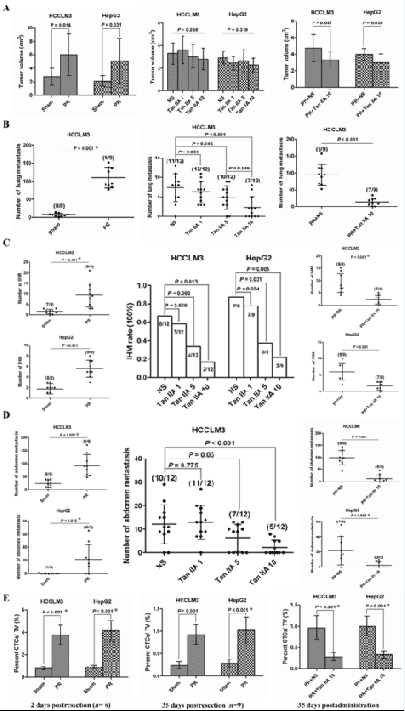
**

**Figure S2. Schematic diagram of intrahepatic and abdomen metastases.** (A) Intrahepatic metastatic lesions were shown by HE staining (50×). (B) Typical abdomen metastasis of residual HCCLM3 tumor 35 d after palliative resection. Panels **a** and **b** show intrahepatic, peritoneal, and diaphragmatic metastatic lesions. **c** and **d** show intrahepatic and diaphragmatic metastatic lesions; **d** shows the area where the tumor in **c** was removed.

**
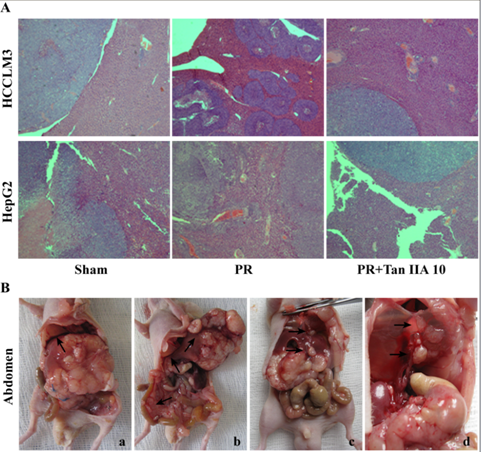
**

**Figure S3. Monitoring body weight of experimental mice in three *in vivo* experiments.** (A) In vitro experiment 1 (IE 1). (B) IE (2). (C) IE (3).


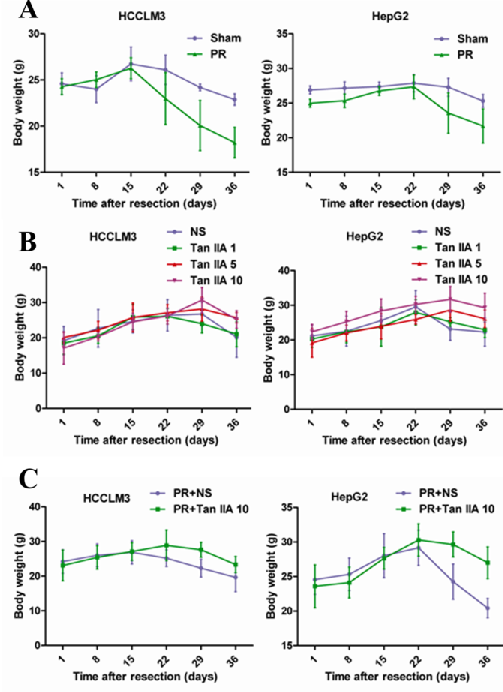


**Figure S4. Intratumoral and intracellular mRNA levels of HIF-1α, N-cadherin, E-cadherin, and Vimentin.** (A) ^*^Compared with Sham group, ^**^compared with PR + NS group; *p*<0.05. (B) ^*^Compared with dimethylsulfoxide (DMSO) group, ^**^compared with hypoxia group; *p*<0.05. (C) ^*^Compared with DMSO group; *p*<0.05.


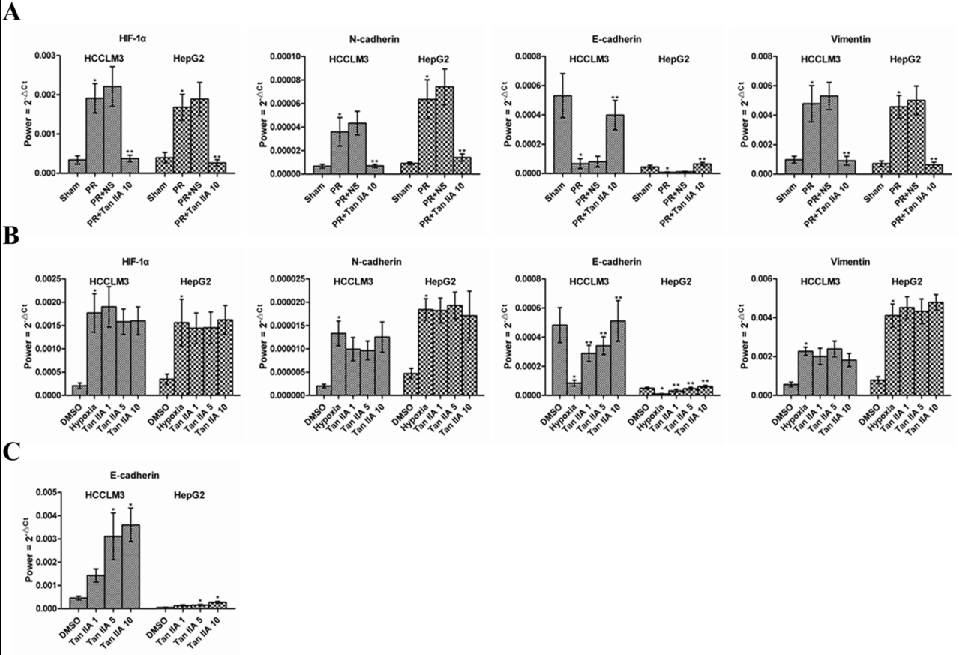


**Figure S5. Immunohistochemical staining of CD31 (A) and NG2 (B) in tumor samples.**

**
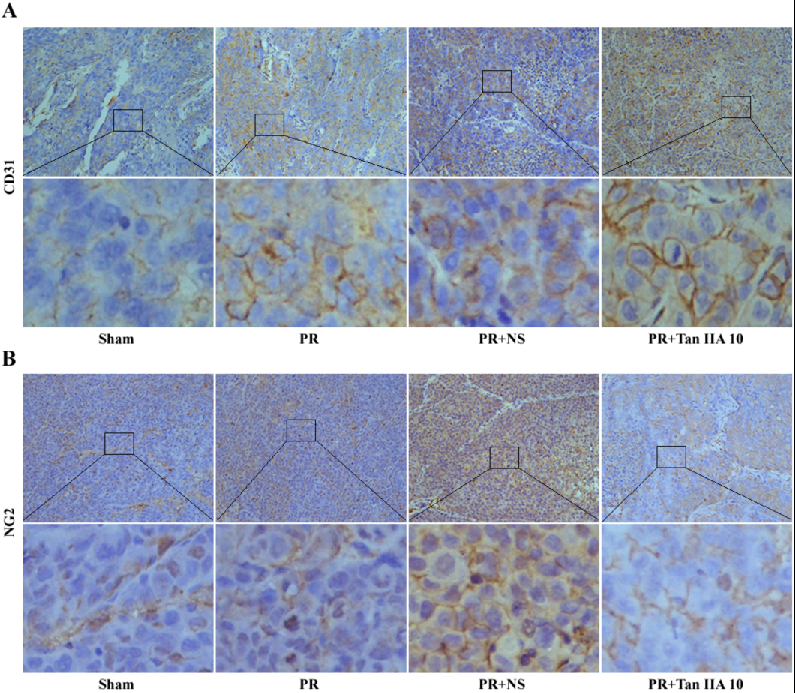
**
